# Association between childhood friendship and cognitive ageing trajectory in later life: evidence from the China Health and Retirement Longitudinal Study (CHARLS)

**DOI:** 10.1186/s12877-022-03181-6

**Published:** 2022-06-09

**Authors:** Jinzhao Xie, Xiaoyan Fan, Ping Yin, Jing Gu, Chengwu Yang

**Affiliations:** 1 Department of Medical Statistics, School of Public Health, Sun Yat-sen University, Guangzhou, China; 2 School of Public Health, LKS Faculty of Medicine, The University of Hong Kong, Hong Kong Special Administrative Region, China; 3 Sun Yat-sen Global Health Institute, School of Public Health and Institute of State Governance, Sun Yat-sen University, Guangzhou, China; 4 Key Laboratory of Health Informatics of Guangdong Province, Sun Yat-sen University, Guangzhou, China; 5 Division of Biostatistics and Health Services Research, Department of Population and Quantitative Health Sciences, UMass Chan Medical School, Worcester, Massachusetts USA 01655; 6 Department of Obstetrics and Gynecology, UMass Chan Medical School, Worcester, Massachusetts USA 01655

**Keywords:** Cognitive ageing, Childhood friendship, Multilevel model, China

## Abstract

**Background:**

Childhood experience has been suggested to affect cognitive function in later life. However, the association between childhood friendship status and cognitive ageing trajectory in middle-aged and older adults has not been fully assessed. This study examined the association between childhood friendship status and cognitive ageing trajectory and identified factors modifying this association.

**Methods:**

We used four waves of data from the China Health and Retirement Longitudinal Study (CHARLS), a national representative longitudinal study of adults aged 45 years or older, 2011–2018. The CHARLS included surveys on childhood friendship and cognitive assessments. Childhood friendship status was categorised as poor, fair, and good. To examine the association between childhood friendship and cognitive ageing trajectory in later life, we applied multilevel linear regression models, and explored potential influences of sociodemographic factors, health status and behaviours, and childhood conditions on this association.

**Results:**

Of the 4,350 participants, 1,919 (44.1%) were women. The mean age was 56.29 ± 7.80 years. We found childhood friendship was significantly associated with cognitive ageing trajectory in later life, with a dose–response relationship. After adjusting for covariates, comparing to participants with poor childhood friendships, those with better childhood friendships had lower rates of cognitive decline (β = 0.12, 95% confidence interval [CI]: 0.03 to 0.22 [interaction term of fair friendship and time]; β = 0.19, 95% CI: 0.10 to 0.28 [interaction term of good friendship and time]) and higher level of cognitive functions (β = 0.40, 95% CI: 0.22 to 0.58 [fair friendships]; β = 0.61, 95% CI: 0.43 to 0.79 [good friendships]). These associations were stronger for those who were female, less educated, and had experienced more adverse childhood experiences.

**Conclusions:**

Childhood friendship is associated with cognitive ageing in later life. Enhancing childhood friendships can play an important role to promote healthy ageing in the future.

**Supplementary Information:**

The online version contains supplementary material available at 10.1186/s12877-022-03181-6.

## Background

The world’s population is growing older. By 2050, elderly aged 65 or above are projected to account for one-sixth of the global population, with an estimated life expectancy of 77.1 years [1]. As a result of the ageing process, elderly people are susceptible to cognitive impairment, which could lead to substantial social and economic costs for caregivers and society at large [2, 3]. Identifying modifiable risk factors for cognitive ageing and impairment could inform development of healthy ageing intervention and strategies [4]. According to life course theory, early-life experiences have a far-reaching influence across the entire life span [5, 6], which has been corroborated by previous evidence that childhood experience, such as education, family socioeconomic status, and migration at a young age, were associated with cognitive function in later life [7–9]. However, there is a dearth of empirical studies assessing whether and how childhood friendships affect the cognitive ageing trajectory and impairment in middle and old age.

High-quality friendship is characterised by high levels of prosocial behaviour, intimacy, and low levels of conflicts or rivalry [10]. Childhood friendship has direct and indirect effects on children’s social development [10], which could then affect personality and behaviour in adulthood [11]. Therefore, high-quality childhood friendship might be positively associated with cognitive function in later life. Similar to childhood adverse events that exert long-lasting changes in neurobiological system [12, 13], childhood friendships might also have a far-reaching impact on cognitive function with childhood being a critical stage for brain growth and cognitive development. Previous findings have suggested that positive childhood friendship was associated with episodic memory in late life, which is regarded as one domain of cognitive function [14]. However, the measure for childhood friendship was not comprehensive and other domains of the cognitive function, such as mental intactness, were not assessed in these studies [14, 15]. Besides, a wide range of determinants of better cognitive function have been well identified, including higher educational level, better socioeconomic status, and fewer adverse life events, etc. [16–18], which could be regarded as the advantageous resources of cognitive function according to Resource Substitution Theory [19]. The theory suggested that the effects of multiple advantageous resources may substitute for each other, such that the absence of one makes the presence of another more important [19]. Therefore, we presume that the association between childhood friendships and cognitive function in later life may be stronger for those who have fewer advantageous resources of high cognitive function. Nevertheless, few studies have explored the potential moderators of the association between childhood friendship status and cognitive ageing in middle-aged and older adults.

China is one of the most rapidly ageing countries in the world, with more than 10.4 million people currently suffering from cognitive impairment [20]. The China Health and Retirement and Longitudinal Study (CHARLS), a nationally representative longitudinal study of adults aged 45 or older, provides us a unique opportunity to investigate the association between childhood friendship status and the cognitive ageing trajectory in middle-aged and older adults. In addition, we explore whether this association is modified by sociodemographic factors, health status and behaviours, or childhood conditions.

## Methods

### Data and study sample

We used the deidentified data from the CHARLS cohort. The study sample was obtained by four-stage stratified sampling using the probability-proportional-to-size technique. The baseline survey of the CHARLS included 17,708 respondents in 28 provinces and was conducted in 2011. Three follow-up assessments were performed, in 2013, 2015, and 2018. In addition, a life history survey was conducted in 2014. The details of the CHARLS have been published elsewhere [21]. The CHARLS program complied with the principles of the Declaration of Helsinki and received ethical approval from the Peking University Institutional Review Board [21, 22]. All participants in the CHARLS provided written informed consent.

We used the data from the 2011 CHARLS baseline survey, all three follow-up assessments, and the 2014 life history survey. We restricted our sample to 4,350 respondents who met the following criteria: (1) aged ≥ 45 years at baseline, (2) completed cognitive assessments at baseline and all three follow-up timepoints, (3) provided information about childhood friendships in the 2014 life history survey. Figure S1 in supplementary material shows the schematic flow of participant selection for the study.

### Measures

#### Cognitive function

We obtained cognitive function data from baseline and all three follow-up assessments. In line with previous studies, cognitive function was measured by the total cognitive function score, which assesses two dimensions: episodic memory and mental intactness [9, 23, 24]. Episodic memory was assessed using word recall tests [9, 23, 24]. In the word recall tests, the investigators read a list of 10 Chinese nouns to the respondents. The respondents were then asked to repeat the word list in any order immediately (immediate recall) and recall the same words 4 min later (delayed recall). The episodic memory score was calculated as the average of the immediate and delayed recall scores [9, 23, 24]. Scores ranged from 0 to 10, with a higher memory score indicating better episodic memory. Mental intactness was assessed using ten items from the Telephone Interview of Cognitive Status test and a pentagon-drawing test [25]. The mental intactness score (range: 0–11) was reported based on the number of successful completions of the following tasks: serial subtraction of 7 from 100 (up to five times); identifying the date (month, day, and year), the day of the week, and the season of the year; and redrawing a picture of two overlapping pentagons. The total cognitive function score was the sum of the episodic memory and mental intactness scores. The scores ranged from 0 to 21, with a higher score indicating better cognitive function [9, 23, 24].

#### Childhood friendship

We obtained information on childhood friendships from the 2014 life history survey in the CHARLS. Childhood friendship was measured based on answers to three questions [15]. The responses to each question were dichotomized, with a score of 1 representing positive childhood friendships and a score of 0 representing negative childhood friendships. The questions and corresponding scores were as follows: “When you were a child, how often did you feel lonely for not having friends (0 = often or sometimes, 1 = not very often or never)”, “Did you often have a group of friends that you felt comfortable spending time with? (1 = often or sometimes, 0 = not very often or never)”, and “Did you have a good friend? (1 = yes, 0 = no)”. The total childhood friendship score was the sum of the score for each question, ranging from 0 to 3. We categorised those who scored 0 and 1 into one group as only one hundred and twenty-two participants scored 0 for childhood friendship. We categorised the participants into the following three groups based on their childhood friendship scores: poor (0 or 1 point), fair (2 points), and good (3 points).

#### Covariates

We investigated sociodemographic variables and childhood conditions in our study. Sociodemographic variables were obtained from the baseline survey and comprised age, sex, residence (rural or urban), marital status (married/partnered or other), educational level, and current household expenditure per capita. Due to relatively low educational level in Chinese middle-aged and older adults (33.41% of participants had an educational level higher than primary school), we categorised educational level into four groups: illiterate, some primary school, finished primary school, and higher than primary school [9, 26–29]. Previous studies suggested that household expenditure per capita had a stronger association with personal well-being and living standards, compared to household income, and therefore was adopted in our analysis to reflect economic status [30, 31]. We divided household expenditure per capita into three levels (low, medium, or high) according to the lower and upper quartiles.

Information on childhood conditions was obtained from the 2014 life history survey. Childhood conditions were determined by four factors: family financial situation (about average, better off, or worse off than the other households in the same community/village), childhood *hukou* status (the household registration system in China, categorised as non-agricultural or agricultural, with non-agricultural representing higher Social-Economic Status, SES), self-reported childhood health (about average, healthier, or less healthy than children of the same age), and the number of adverse childhood experiences. A total of ten adverse childhood experiences were assessed such as family bullying, domestic violence, parental death, incarcerated household members. We categorised respondents into four groups based on the number of cumulative ACEs: 0, 1, 2, and 3 or more. Additional details of covariates are available in supplemental Methods and Table S1 in the Supplementary Material.

### Statistical analysis

Table 1 shows the baseline characteristics of the sample, stratified by childhood friendship status. We assessed the cognitive function trajectory using multilevel linear regression models in which follow-up wave was set as the first level (level 1) and coded as 0, 1, 2, and 3 to represent the longitudinal term, and individuals were set as the second level (level 2) to account for between individual variation.

**Table 1:** Baseline characteristics of participants by childhood friendship status

Characteristics	Total (*n* = 4,350)	Childhood friendship, No. (%)			*p* value
		Poor (*n* = 692)	Fair (*n* = 1,540)	Good (*n* = 2,118)	
**Sociodemographic**					
Age, mean (SD)	56.29 (7.80)	58.37 (7.92)	56.71 (7.69)	55.31 (7.68)	< 0.001
Female	1919 (44.1)	274 (39.6)	651 (42.3)	994 (46.9)	0.001
Rural residence	2501 (57.5)	480 (69.4)	922 (59.9)	1099 (51.9)	< 0.001
Married/partnered	4041 (92.9)	630 (91.0)	1437 (93.3)	1974 (93.2)	0.116
Educational level					
Illiterate	277 (6.4)	73 (10.5)	110 (7.1)	94 (4.4)	< 0.001
Some primary school	657 (15.1)	172 (24.9)	250 (16.2)	235 (11.1)	
Finished primary school	1160 (26.7)	207 (29.9)	456 (29.6)	497 (23.5)	
Higher than primary school	2256 (51.9)	240 (34.7)	724 (47.0)	1292 (61.0)	
Household expenditure per capita					
Low	805 (21.1)	158 (25.6)	302 (22.7)	345 (18.5)	< 0.001
Medium	1943 (51.0)	324 (52.6)	711 (53.5)	908 (48.7)	
High	1060 (27.8)	134 (21.8)	316 (23.8)	610 (32.7)	
**Childhood conditions**					
Childhood family financial situation					
Worse off	1602 (36.9)	327 (47.3)	595 (38.7)	680 (32.1)	< 0.001
About average	2311 (53.2)	327 (47.3)	823 (53.5)	1161 (54.8)	
Better off	434 (10.0)	37 (5.4)	121 (7.9)	276 (13.0)	
Childhood hukou (non-agricultural)	519 (12.0)	46 (6.7)	140 (9.1)	333 (15.8)	< 0.001
Childhood health					
Less healthy	494 (11.4)	108 (15.6)	174 (11.3)	212 (10.0)	< 0.001
About average	2164 (49.8)	357 (51.7)	810 (52.7)	997 (47.1)	
Healthier	1689 (38.9)	226 (32.7)	554 (36.0)	909 (42.9)	
Adverse childhood experiences					
0	1240 (28.5)	156 (22.5)	403 (26.2)	681 (32.2)	< 0.001
1	1525 (35.1)	232 (33.5)	546 (35.5)	747 (35.3)	
2	957 (22.0)	178 (25.7)	360 (23.4)	419 (19.8)	
≥ 3	628 (14.4)	126 (18.2)	231 (15.0)	271 (12.8)	
**Cognitive function**					
Total cognitive score, mean (SD)	13.40 (2.67)	12.67 (2.80)	13.26 (2.59)	13.74 (2.61)	< 0.001
Mental intactness score, mean (SD)	9.29 (1.80)	8.90 (1.97)	9.22 (1.77)	9.47 (1.74)	< 0.001
Episodic memory score, mean (SD)	4.11 (1.55)	3.76 (1.54)	4.03 (1.55)	4.27 (1.54)	< 0.001

*SD* standard deviation

We constructed a series of models to adjust for different sets of confounding factors. Model 1 only adjusted for age and sex. In Model 2, we added the interaction term of time and childhood friendship status, based on Model 1, to explore the association between childhood friendship status and the rate of decline in cognitive function. To explore the effects of different sets of confounding factors on the association between childhood friendship status and cognitive function decline, we added sociodemographic variables and childhood conditions to Models 3 and 4 sequentially. We performed trend tests to investigate the possible dose–response relationship by modelling the ordered childhood friendship status as a one degree-of-freedom linear term [32]. As a supplementary analysis, we repeated the above procedures without the interaction term of time and childhood friendship status to assess the association between childhood friendship status and the level of cognitive function. To identify the moderators of the association between childhood friendship status and cognitive function decline, we assessed the three-way interaction terms of childhood friendship status, time and each covariate in separate models, while adjusting for the remaining covariates. We also evaluated the influence of moderators on the association between childhood friendship status and the level of cognitive function by assessing interaction terms of childhood friendship status and each covariate as a supplementary analysis.

Since only participants with complete data on cognitive function in all follow up waves were included in the analysis, we compared the baseline characteristics between participants included and not included in our study (Table S2). In sensitivity analysis, we used multiple imputation by chained equations (MICE) to impute missing values for those who did not have completed cognitive assessments during the follow-up surveys, and a total of 13,333 participants were included in imputed dataset. We replicated the main analyses based on imputed dataset, and achieved similar results (Table S3). For all analyses, we used the R software package, version 4.1.1 [33]. Multilevel linear models and multiple imputation were performed by using ‘lme4’ and ‘mice’ package respectively. Statistical significance was based on a two-tailed *p* value < 0.05.

## Results

### Baseline characteristics

Of the 4,350 participants included in the analysis (mean [standard deviation] age 56.29 [7.80] years; 44.1% women), 692 (15.9%), 1,540 (35.4%), and 2,118 (48.7%) had poor, fair, and good childhood friendships, respectively. Most of the participants were married (92.9%) and had had at least one ACE (71.5%). Approximately half of the participants had an educational level higher than primary school (51.9%), lived in a rural area (57.5%), had a medium current household expenditure per capita (51.0%), had an average childhood family financial situation (53.2%), and had average childhood health (49.8%). The average total cognitive score was 13.40 ± 2.67. Compared to participants who had good childhood friendships, those who had poor childhood friendships were more likely to be older, male, residents of a rural area, less educated and have a lower household consumption per capita, experienced a worse childhood family financial situation, an agriculture *hukou* status, experienced worse childhood health, had more ACEs, and a lower total cognitive score (Table 1). The scores of total cognitive and mental intactness showed a decline tendency during follow-up period. Mean scores of cognitive measures from 2011 to 2018 by childhood friendship status were present in Figure S2 in additional file.

### Association between childhood friendship status and cognitive ageing trajectory in later life

**Fig. 1:**
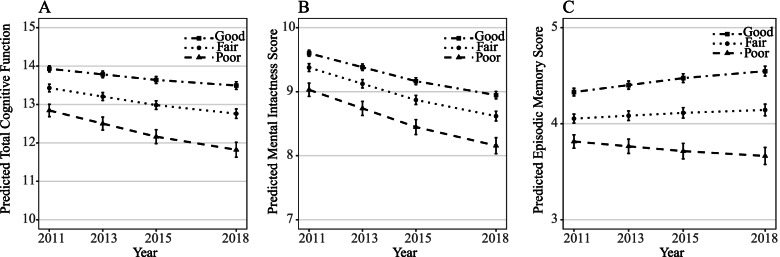
Predicted cognitive ageing trajectories by childhood friendship status. **A** Predicted total cognitive function scores by childhood friendship status. **B** Predicted mental intactness scores by childhood friendship status. **C** Predicted episodic memory scores by childhood friendship status

Table 2 presents the results of a multilevel regression analysis with the interaction term of childhood friendship status and time included, except in Model 1. After adjusting for covariates, the coefficients of time in the models for total cognitive function and mental intactness are negative, indicating that total cognitive function and mental intactness declined with time. For the total cognitive score, the coefficients of the interaction terms of childhood friendship status (fair or good) and time are consistently positive in Models 2 to 4, indicating that those who had had fair or good childhood friendships showed a slower decline in total cognitive function in middle or late age than those who had not. A dose–response relationship is observed between childhood friendship status and cognitive decline, which indicates that individuals with better childhood friendships showed a slower decline in cognitive function in middle and old age than those who had worse childhood friendships. Similar results are observed for mental intactness and episodic memory (Table 2). The predicted cognitive function scores based on models adjusted for all covariates were presented in Fig. 1.

**Table 2:** Association between childhood friendship status and cognitive ageing trajectory in middle-aged and older adults

Fixed effect	β (95% CI)	Model 2^b^	Model 3^c^	Model 4^d^
	Model 1^a^			
**Total cognitive function**				
Constant	15.37 (14.86, 15.88)^***^	15.54 (15.01, 16.07)^***^	12.00 (11.33, 12.67)^***^	12.20 (11.49, 12.90)^***^
Time	-0.21 (-0.23, -0.19)^***^	-0.36 (-0.44, -0.28)^***^	-0.36 (-0.44, -0.28)^***^	-0.35 (-0.43, -0.27)^***^
Childhood friendship (Ref: poor)				
Fair	0.71 (0.51, 0.91)^***^	0.54 (0.32, 0.76)^***^	0.26 (0.04, 0.48)^*^	0.25 (0.03, 0.46)^*^
Good	1.23 (1.05, 1.41)^***^	1.00 (0.78, 1.22)^***^	0.40 (0.18, 0.62)^***^	0.37 (0.16, 0.58)^***^
Childhood friendship (Ref: poor) × Time				
Childhood friendship (fair) × Time	–	0.15 (0.05, 0.25)^**^	0.13 (0.03, 0.23)^**^	0.12 (0.03, 0.22)^*^
Childhood friendship (good) × Time	–	0.21 (0.13, 0.29)^***^	0.19 (0.09, 0.29)^***^	0.19 (0.10, 0.28)^***^
*p* value for trend	–	< 0.001	< 0.001	< 0.001
**Mental intactness**				
Constant	9.48 (9.15, 9.81)^***^	9.55 (9.22, 9.88)^***^	7.20 (6.75, 7.65)^***^	7.27 (6.80, 7.74)^***^
Time	-0.24 (-0.26, -0.22)^***^	-0.29 (-0.35, -0.23)^***^	-0.29 (-0.35, -0.23)^***^	-0.29 (-0.34, -0.23)^***^
Childhood friendship (Ref: poor)				
Fair	0.39 (0.27, 0.51)^***^	0.33 (0.19, 0.47)^***^	0.17 (0.03, 0.31)^*^	0.16 (0.02, 0.31)^*^
Good	0.67 (0.55, 0.79)^***^	0.59 (0.45, 0.73)^***^	0.24 (0.10, 0.38)^***^	0.23 (0.09, 0.37)^**^
Childhood friendship (Ref: poor) × Time				
Childhood friendship (fair) × Time	–	0.05 (-0.01, 0.11)^†^	0.05 (-0.01, 0.11)	0.04 (-0.02, 0.10)
Childhood friendship (good) × Time	–	0.07 (0.01, 0.13)^*^	0.07 (0.01, 0.13)^*^	0.06 (0.00, 0.12)^*^
*p* value for trend	–	0.024	0.038	0.044
**Episodic memory**				
Constant	6.00 (5.73, 6.27)^***^	6.12 (5.85, 6.39)^***^	4.85 (4.48, 5.22)^***^	4.97 (4.58, 5.37)^***^
Time	0.03 (0.01, 0.05)^**^	-0.07 (-0.11, -0.03)^**^	-0.07 (-0.13, -0.01)^**^	-0.07 (-0.12, -0.02)^*^
Childhood friendship (Ref: poor)				
Fair	0.33 (0.23, 0.43)^***^	0.21 (0.09, 0.33)^***^	0.08 (-0.06, 0.22)	0.08 (-0.05, 0.21)
Good	0.57 (0.47, 0.67)^***^	0.40 (0.28, 0.52)^***^	0.15 (0.03, 0.27)^*^	0.13 (0.01, 0.26)^*^
Childhood friendship (Ref: poor) × Time				
Childhood friendship (fair) × Time	–	0.10 (0.04, 0.16)^***^	0.09 (0.03, 0.15)^**^	0.08 (0.02, 0.14)^**^
Childhood friendship (good) × Time	–	0.14 (0.08, 0.20)^***^	0.13 (0.07, 0.19)^***^	0.13 (0.07, 0.19)^***^
*p* value for trend	–	< 0.001	< 0.001	< 0.001

^†^0.05 ≤ *p* < 0.1; ^*^p < 0.05; ^**^p < 0.01; ^***^p < 0.001

–: Not included in model

^a^Model 1 was adjusted for age and sex

^b^Model 2 was adjusted as per Model 1 plus with the interaction term of time and childhood friendship

^c^Model 3 was adjusted as per Model 2 plus with sociodemographic factors (residence, marital status, educational level, and household expenditure per capita)

^d^Model 4 was adjusted as per Model 3 plus with childhood conditions (childhood family financial situation, childhood first hukou, childhood health, and adverse childhood experiences)

Additional analyses based on models without the interaction term of time and childhood friendship status show that those who had better childhood friendships were more likely to have a higher cognitive function score than those who had not (Table S4). For the total cognitive function score, the coefficient of childhood friendship status remains consistently positive in Models 1 to 3. A dose–response relationship was also observed. The coefficient for good childhood friendship decreased by 50.4% from model 1 (1.23, 95% confidence interval [CI]: 1.05–1.41) to model 3 (0.61, 95% CI: 0.43–0.79), which indicated the additional adjustment of the covariates (i.e., sociodemographic variables and childhood conditions) in model 3 explained 50.4% of the variation in total cognitive function level between those with good and those with poor childhood friendships. Similar results were observed for mental intactness and episodic memory (Table S4).

### Influences of covariates on the association between childhood friendship status and cognitive function in later life

Factors that modify the association between childhood friendship status and cognitive function decline are presented in Table 3. For total cognitive function, after adjusting for covariates, the interaction terms of good childhood friendship, time and experienced ACEs are significantly positive (β = 0.33, 95% CI: 0.09 to 0.57 [childhood friendship (good) × time × ACEs (1)]; β = 0.34, 95% CI: 0.09 to 0.60 [childhood friendship (good) × time × ACEs (2)]). This result indicated that the association between childhood friendship status and cognitive function decline was stronger among those who experienced one or two adverse childhood events compared to those who had no childhood adversities. Similar results were observed for mental intactness and episodic memory (Table 3).

**Table 3:** Interaction effects of potential moderators and childhood friendship status on cognitive decline in middle-aged and older adults

Model	Total cognitive function, β (95% CI)	Mental intactness, β (95% CI)	Episodic memory, β (95% CI)
**Model 1**^**a**^			
Time	-0.42 (-0.52, -0.32)^***^	-0.30 (-0.37, -0.24)^***^	-0.11 (-0.18, -0.05)^***^
Childhood friendship (Ref: poor)			
Fair	0.09 (-0.19, 0.36)	0.09 (-0.10, 0.27)	0.00 (-0.17, 0.16)
Good	0.26 (-0.02, 0.53)^†^	0.14 (-0.04, 0.32)	0.10 (-0.06, 0.27)
Female (Ref: male)	-0.53 (-0.89, -0.16)^**^	-0.59 (-0.83, -0.35)^***^	0.06 (-0.16, 0.28)
Childhood friendship (fair) × Time	0.17 (0.04, 0.29)^**^	0.05 (-0.03, 0.13)	0.12 (0.04, 0.20)^**^
Childhood friendship (good) × Time	0.19 (0.07, 0.30)^**^	0.04 (-0.04, 0.12)	0.15 (0.07, 0.22)^***^
Childhood friendship (fair) × Time × Female	-0.11 (-0.30, 0.08)	-0.02 (-0.15, 0.11)	-0.09 (-0.21, 0.03)
Childhood friendship (good) × Time × Female	-0.02 (-0.20, 0.17)	0.04 (-0.08, 0.16)	-0.06 (-0.17, 0.06)
**Model 2**^**a**^			
Time	-0.54 (-0.78, -0.29)^***^	-0.33 (-0.50, -0.17)^***^	-0.21 (-0.36, -0.05)^*^
Childhood friendship (Ref: poor)			
Fair	0.92 (0.20, 1.63)^*^	0.70 (0.22, 1.18)^**^	0.22 (-0.21, 0.65)
Good	0.74 (0.01, 1.48)^*^	0.46 (-0.04, 0.95)^†^	0.29 (-0.15, 0.73)
Educational level (Ref: illiterate)			
Some primary school	1.93 (1.27, 2.59)^***^	1.40 (0.96, 1.84)^***^	0.54 (0.14, 0.93)^**^
Finished primary school	2.26 (1.62, 2.91)^***^	1.71 (1.28, 2.15)^***^	0.55 (0.17, 0.94)^**^
Higher than primary school	3.07 (2.44, 3.71)^***^	2.29 (1.86, 2.72)^***^	0.78 (0.40, 1.16)^***^
Childhood friendship (fair) × Time	-0.12 (-0.44, 0.19)	-0.03 (-0.24, 0.18)	-0.09 (-0.30, 0.11)
Childhood friendship (good) × Time	0.09 (-0.23, 0.41)	0.09 (-0.13, 0.31)	0.00 (-0.21, 0.21)
Childhood friendship (fair) × Time × Some primary school	0.29 (-0.08, 0.66)	0.10 (-0.16, 0.35)	0.20 (-0.04, 0.43)
Childhood friendship (fair) × Time × Finished primary school	0.22 (-0.14, 0.57)	0.07 (-0.17, 0.31)	0.15 (-0.08, 0.37)
Childhood friendship (fair) × Time × Higher than primary school	0.24 (-0.10, 0.59)	0.07 (-0.16, 0.31)	0.17 (-0.05, 0.40)
Childhood friendship (good) × Time × Some primary school	-0.04 (-0.42, 0.34)	-0.14 (-0.40, 0.12)	0.10 (-0.14, 0.35)
Childhood friendship (good) × Time × Finished primary school	0.00 (-0.36, 0.36)	-0.06 (-0.30, 0.19)	0.06 (-0.18, 0.29)
Childhood friendship (good) × Time × Higher than primary school	0.10 (-0.25, 0.45)	0.00 (-0.24, 0.24)	0.10 (-0.13, 0.33)
**Model 3**^**a**^			
Time	-0.19 (-0.36, -0.03)^*^	-0.21 (-0.32, -0.09)^***^	0.01 (-0.10, 0.12)
Childhood friendship (Ref: poor)			
Fair	0.62 (0.18, 1.06)^**^	0.31 (0.01, 0.60)^*^	0.31 (0.05, 0.58)^*^
Good	0.53 (0.11, 0.95)^*^	0.23 (-0.05, 0.51)	0.29 (0.04, 0.54)^*^
Adverse childhood experiences (Ref: 0)			
1	0.13 (-0.35, 0.62)	0.03 (-0.29, 0.36)	0.10 (-0.19, 0.39)
2	0.30 (-0.21, 0.81)	0.04 (-0.30, 0.39)	0.26 (-0.05, 0.56)
≥ 3	-0.20 (-0.76, 0.35)	-0.39 (-0.76, -0.01)^*^	0.19 (-0.15, 0.52)
Childhood friendship (fair) × Time	-0.03 (-0.22, 0.16)	-0.07 (-0.20, 0.06)	0.04 (-0.08, 0.17)
Childhood friendship (good) × Time	-0.03 (-0.21, 0.15)	-0.06 (-0.18, 0.06)	0.03 (-0.09, 0.15)
Childhood friendship (fair) × Time × ACEs (1)	0.30 (0.04, 0.55)^*^	0.22 (0.05, 0.39)^**^	0.07 (-0.09, 0.24)
Childhood friendship (fair) × Time × ACEs (2)	0.11 (-0.16, 0.38)	0.07 (-0.11, 0.25)	0.04 (-0.13, 0.22)
Childhood friendship (fair) × Time × ACEs (≥ 3)	0.13 (-0.17, 0.43)	0.12 (-0.08, 0.32)	0.01 (-0.18, 0.20)
Childhood friendship (good) × Time × ACEs (1)	0.33 (0.09, 0.57)^**^	0.24 (0.08, 0.40)^**^	0.10 (-0.06, 0.25)
Childhood friendship (good) × Time × ACEs (2)	0.34 (0.09, 0.60)^**^	0.16 (-0.02, 0.33)^†^	0.19 (0.02, 0.35)^*^
Childhood friendship (good) × Time × ACEs (≥ 3)	0.16 (-0.12, 0.45)	0.03 (-0.16, 0.22)	0.13 (-0.05, 0.32)

ACEs, adverse childhood experiences

^†^0.05 ≤ *p* < 0.1; ^*^p < 0.05; ^**^p < 0.01; ^***^p < 0.001

^a^Model was adjusted for sociodemographic factors and childhood conditions

Models for cognitive function with the interaction terms of childhood friendship status and each covariate are presented in Table S5. The association between childhood friendship status and the level of mental intactness were stronger for those who were male, were less educated, and had experienced ACEs (Table S5). Factors modifying the association between childhood friendship status and episodic memory are not observed.

## Discussion

In this national longitudinal study in China, we found a positive association between childhood friendship status and cognitive ageing trajectory in middle-aged and older individuals. This finding shows that those who had better childhood friendships are more likely have a slower rate of cognitive decline and a higher level of cognitive function in middle and old age than those who had had worse childhood friendships. Specifically, we found that the association of childhood friendship status with cognitive function was stronger for those who were female, less educated, and had had more ACEs.

Our findings suggest that better childhood friendship status is associated with better cognitive function and slower cognitive ageing process in later life. This finding is consistent with previous evidence supporting the importance of childhood as the crucial period of cognitive function development [34]. Longitudinal studies have found that better childhood friendship status was negatively associated with psychological difficulties in young adulthood and depressive symptoms in middle and old age [35, 36]. Our findings add to the body of evidence on the long-lasting influence of positive childhood experiences on the development of cognitive function. This highlights that extra efforts should be made to help children build friendships at an early age. As a group meeting-based intervention was shown to improve the social skills and social competence of children who have experienced peer relationship difficulties, such interventions should be further evaluated and implemented in real-world settings [37].

Moreover, we identified factors, such as being female and less educated, and having experienced more ACEs (i.e., one or two adverse events), that modify the association of childhood friendship status with cognitive function. According to resource substitution theory, the effect of having a specific advantageous resource is greater for groups that have fewer alternatives. Individuals that are female, less educated, and having experienced one or two ACEs (as compared to those experienced no ACEs) have fewer advantageous resources of cognitive function, and thus would benefit more from having better childhood friendships. Women are more likely to have worse or more rapidly declining cognitive function than men, which may be due to longer periods of domestic work, less engagement in social activities, and less formal education [38, 39]. Therefore, women may have more emotional gain from having good childhood friendships than men. Similarly, those who have a lower educational level, and have had more ACEs may be more sensitive to the effect of childhood friendship status on cognition given their lack of alternative advantageous resources [40]. Early-age psychosocial interventions targeting young adults should be prioritized for individuals with these characteristics. However, most variables assessed in our analysis were not found to modify the association between childhood friendship status and episodic memory. According to Cattell’s psychometrically based theory, adult mental capacity consists of fluid intelligence and crystallized intelligence, with the former referring to the ability to solve novel reasoning problems [41]. Fluid intelligence, commonly seen as having a strong hereditary component, has been suggested to be robust against influence of education and socialisation [42]. Given previous studies suggested that memory is closely related to fluid ability [43], the absence of effect moderators could be reflective of the steadiness of episodic memory.

This study has several limitations. First, given our observational study design, we cannot rule out the possibility of reverse causality. Although our assumption regarding the causality between childhood friendship status and cognitive function is supported by the life course theory, our findings are subject to reverse causality and should be interpreted with caution [44]. Second, childhood friendship in our study was measured in middle- and old- age, and hence was subject to recall bias. Given the lack of longitudinal studies that span from childhood to elderly in China, our current findings would be valuable when assessing the influence of childhood experience on later life. Third, our main analysis only included those who completed baseline and all three follow-up surveys to measure the cognitive ageing trajectory. Although participants lost to follow-up had different characteristics compared to those who completed the study, the results remain consistent in our sensitivity analysis using imputed data. Last, although we combined three items to measure childhood friendship status, additional details about childhood friendship were not measured in CHARLS. Further studies that explore the effects of various patterns of childhood friendship on cognition in later life are warranted.

## Conclusions

Based on the national longitudinal study in China, we found that better childhood friendship was associated with better cognitive function and slower decline of cognitive ageing process in later life. The association between childhood friendship and cognitive function was stronger among those who were female, less educated, and had experienced more ACEs. These findings highlight the needs of improving childhood friendships to promote healthy ageing, and the needs of tailored interventions for middle-aged and older adults who have had poor childhood friendships. These preventive measures can slow down cognitive impairment in later life, thus ease social and economic burdens to the ageing world in the future.

## Supplementary Information

**Additional file 1: Figure S1.** Flowchart of participant selection. **Figure S2.** Mean scores of cognitive measures from 2011 to 2018 by childhood friendship status. **Supplemental Methods**. **Table S1.** Adverse childhood experiences (ACEs) and it’s questionnaire items. **Table S2.** Baseline characteristics between participants included and not included. **Table S3.** Association between childhood friendship status and cognitive ageing trajectory in middle-aged and older adults in imputed dataset. **Table S4.** Association between childhood friendship and the level of cognitive function among middle-aged and older adults. **Table S5.** Interaction effects of childhood friendship status and potential moderators on the level of cognitive function in middle-aged and older adults.

## Data Availability

The CHARLS datasets, which analysed during the current study, are publicly available at the National School of Development, Peking University (http://charls.pku.edu.cn/en) and can be obtained after submitting a data use agreement to the CHARLS team.

## Abbreviations

ACEs: Adverse childhood experiences
ADL: Activities of daily living
CHARLS: The China Health and Retirement Longitudinal Study
CI: Confidence interval
SD: Standard deviation
SES: Social-economic status
TICS: Telephone Interview for Cognitive Status
