# Supplementary material for: Association between childhood friendship and cognitive ageing trajectory in later life: evidence from the China Health and Retirement Longitudinal Study (CHARLS)
Source: BMC Geriatr. 2022 Jun 9;22:494. doi: 10.1186/s12877-022-03181-6 (PMC9178862; doi:10.1186/s12877-022-03181-6)
Supplement: Supplementary file 1 — Additional file 1: Figure S1. Flowchart of participant selection. Figure S2. Mean scores of cognitive measures from 2011 to 2018 by childhood friendship status. Supplemental Methods. Table S1. Adverse childhood experiences (ACEs) and it’s questionnaire items. Table S2. Baseline characteristics between participants included and not included. Table S3. Association between childhood friendship status and cognitive ageing trajectory in middle-aged and older adults in imputed dataset. Table S4. Association between childhood friendship and the level of cognitive function among middle-aged and older adults. Table S5. Interaction effects of childhood friendship status and potential moderators on the level of cognitive function in middle-aged and older adults. [file 12877_2022_3181_MOESM1_ESM.docx]

**Association between Childhood Friendship and Cognitive Ageing Trajectory in Later Life: Evidence from the China Health and Retirement Longitudinal Study (CHARLS)**

**Additional File**

Figure S1. Flowchart of participant selection

Figure S2. Mean scores of cognitive measures from 2011 to 2018 by childhood friendship status

Supplemental Methods

1) Socio-demographic variables

2) Childhood conditions

Supplementary tables

Table S1. Adverse childhood experiences (ACEs) and it’s questionnaire items

Table S2. Baseline characteristics between participants included and not included

Table S3. Association between childhood friendship status and cognitive ageing trajectory in middle-aged and older adults in imputed dataset

Table S4. Association between childhood friendship and the level of cognitive function among middle-aged and older adults

Table S5. Interaction effects of childhood friendship status and potential moderators on the level of cognitive function in middle-aged and older adults

**
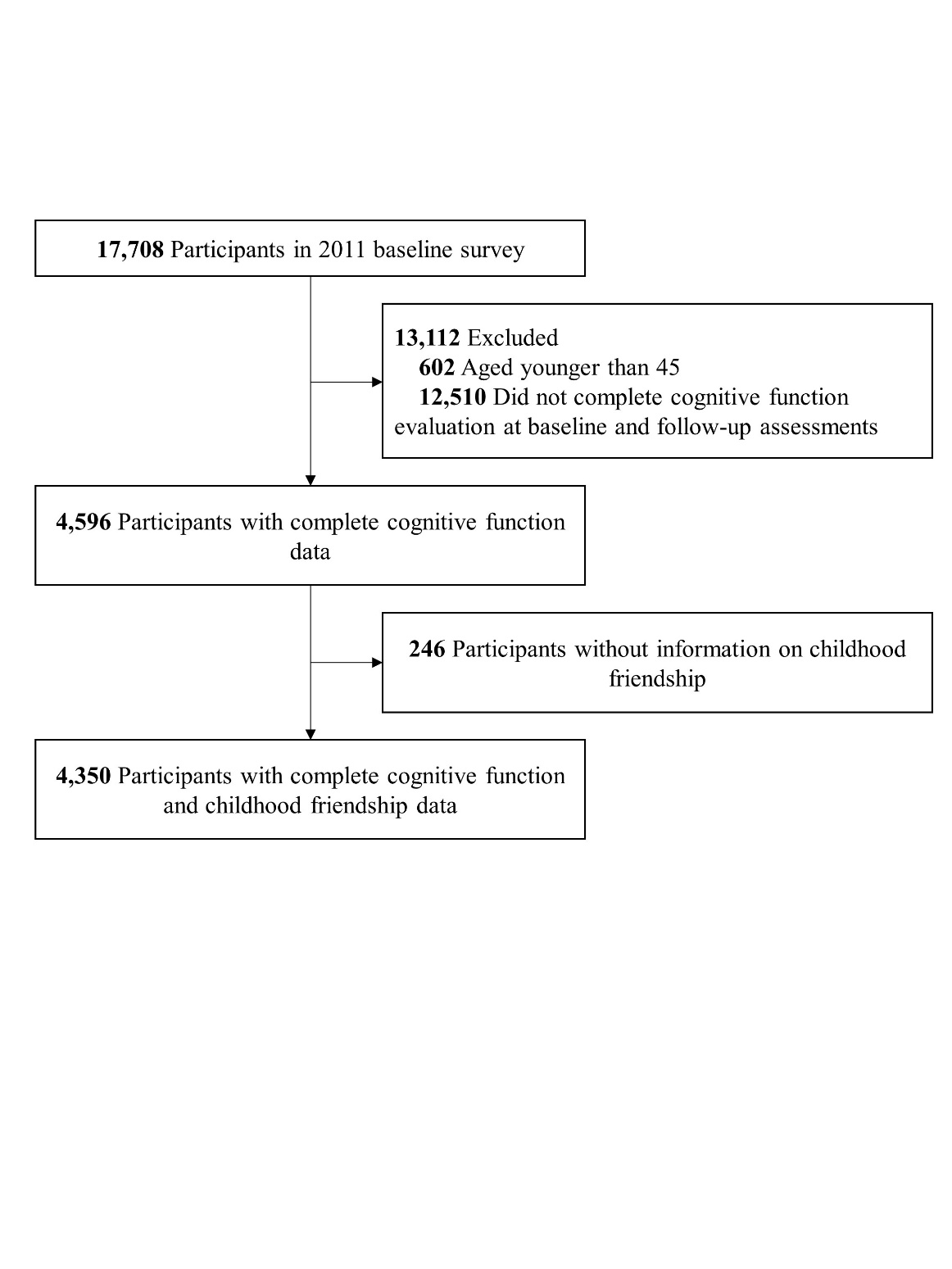
**

**Figure S1. Flowchart of participant selection**

**
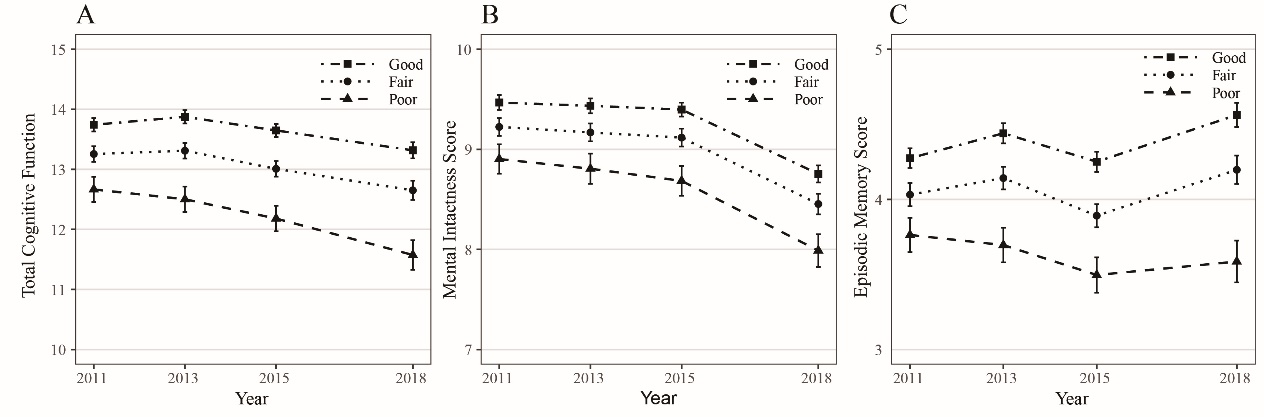
**

**Figure S2. Mean scores of cognitive measures from 2011 to 2018 by childhood friendship status** (A) Total cognitive function scores by childhood friendship status. (B) Mental intactness scores by childhood friendship status. (C) Episodic memory scores by childhood friendship status.

**Supplemental Methods**

**1. Socio-demographic variables**

Residence indicates respondents’ household living region, which was divided into rural or urban based on National Bureau of Statistics of the People's Republic of China.

In CHARLS, the categories of marital status include: 1) married with spouse present, 2) married but not living with spouse temporarily for reasons such as work, 3) separated, 4) divorced, 5) widowed, or 6) never married. Marital status in our study was categorised into married/partnered and others. Married/partnered included the first two statuses, and others indicated the last four statuses.

There were eleven categories of educational levels in CHARLS, including 1) no formal education (illiterate), 2) did not finish primary school but can read, 3) Sishu (private tutoring), 4) elementary/primary school, 5) middle school, 6) high school, 7) vocational school, 8) two-/three-year college/associate degree, 9) four-year college/Bachelor’s degree, 10) Master’s degree, and 11) Doctoral degree/Ph.D. Due to relatively low educational level in Chinese middle-aged and older adults (thirty-three percent of participants had an educational level higher than primary school), we divided educational levels into four levels: illiterate, some primary school, finished primary school and higher than primary school [1-3].

Household consumption per capita was calculated by taking total household consumption divided by the number of people in the household. Total household consumption comprised of a wide range of expenditures, which was the sum of food consumption and non-food consumption (e.g., communication fee, utilities including water and electricity, fuels, fees for housekeeper, matron and servants, local transportation, entertainment, clothing and bedding, long distance traveling expenditure, and medical expenditure). This variable was calculated as annual expenditures. We divided this variable into three levels according to the lower and upper quartiles.

**2. Childhood conditions**

Childhood family financial situation was obtained by the question, “When you were a child before age 17, compared to the average family in the same community/village at that time, how was your family’s financial situation?”. If respondent answered “a lot better off than them” or “somewhat better off than them”, he/she was assigned into “better” group. If respondent answered “somewhat worse off than them” or “a lot worse off than them”, he/she was assigned into “worse” group. Those who chose “the same as them” were assigned into “average” group.

Self-report childhood health was based on the question, “Before you were 15 years old (including 15 years old), would you say that compared to other children of the same age, you were much healthier, somewhat healthier, about average, somewhat less healthy or much less healthy.” Those who chose the first two answers were assigned into “healthier” group. In contrast, those who chose the last two answers were assigned into “less healthy” group. Others were assigned into “about average” group.

The adverse childhood experiences (ACEs) was measure by the number of adverse events respondents experienced in childhood. According to previous study, a total of 10 adverse events were involved in our study, which was presented in Table S1 [1]. We categorised respondents into four groups based on the number of cumulative ACEs: 0, 1, 2, and 3 or more.

**Table S1. Adverse childhood experiences (ACEs) and it’s questionnaire items**

| ACEs | Questionnaire items |
| --- | --- |
| Parental mental health problem | During the years you were growing up, had your female/male guardian showed continued signs of sadness or depression that lasted 2 weeks or more? (yes) |
| Family bullying | When you were growing up, did your female/male guardian ever hit you? (often/sometimes) |
| Parental addiction | During the years you were growing up, did your female/male guardian ever have alcoholism, gambling or drug? (yes) |
| Emotional neglect | How much love and affection did your female guardian give you while you were growing up? (rarely or never) |
| Domestic violence | Have your father/mother ever beat up your mother/father? (often, sometimes) |
| Incarcerated household member | During the years you were growing up, have your female/male guardian ever been arrested or sent to prison? (yes) |
| Parental separation or divorce | Were your biological parents divorced (including long separation due to emotional problems) before you were 17 years? (yes) |
| Parental death | Either of the parents was dead before participant was 17 years. (yes) |
| Sibling death | Any of the siblings was dead before participant was 17 years. (yes) |
| Parental disability | Did your female/male guardian have a long time being sick on bed when you were young? (yes) |

**Table S2. Baseline characteristics between participants included and not included**

| Characteristics | Participants, No. (%) |  | *p* value |
| --- | --- | --- | --- |
|  | Included (n=4350) | Excluded (n=13358) |  |
| **Sociodemographic** |  |  |  |
| Age, mean (SD) | 56.29 (7.80) | 59.22 (10.74) | <0.001 |
| Female | 1919 (44.1) | 7309 (54.7) | <0.001 |
| Rural residence | 2501 (57.5) | 8036 (60.2) | 0.002 |
| Married/partnered | 4041 (92.9) | 11407 (85.5) | <0.001 |
| Educational level |  |  | <0.001 |
| Illiterate | 277 (6.4) | 4574 (34.3) |  |
| Some primary school | 657 (15.1) | 2441 (18.3) |  |
| Finished primary school | 1160 (26.7) | 2662 (20.0) |  |
| Higher than primary school | 2256 (51.9) | 3665 (27.5) |  |
| Household expenditure per capita |  |  | <0.001 |
| Low | 805 (21.1) | 2884 (26.3) |  |
| Medium | 1943 (51.0) | 5445 (49.7) |  |
| High | 1060 (27.8) | 2632 (24.0) |  |
| **Childhood conditions** |  |  |  |
| Childhood family financial situation |  |  | <0.001 |
| Worse off | 1602 (36.9) | 4129 (41.4) |  |
| About average | 2311 (53.2) | 4986 (50.0) |  |
| Better off | 434 (10.0) | 858 (8.6) |  |
| Childhood hukou (non-agricultural) | 519 (12.0) | 737 (7.3) | <0.001 |
| Childhood health |  |  | <0.001 |
| Less healthy | 494 (11.4) | 1388 (13.9) |  |
| About average | 2164 (49.8) | 5268 (52.9) |  |
| Healthier | 1689 (38.9) | 3298 (33.1) |  |
| Adverse childhood experiences |  |  | 0.003 |
| 0 | 1240 (28.5) | 2839 (28.1) |  |
| 1 | 1525 (35.1) | 3361 (33.3) |  |
| 2 | 957 (22.0) | 2184 (21.7) |  |
| ≥3 | 628 (14.4) | 1702 (16.9) |  |
| **Cognitive function** |  |  |  |
| Total cognitive score, mean (SD) | 13.40 (2.67) | 11.65 (3.61) | <0.001 |
| Mental intactness score, mean (SD) | 9.29 (1.80) | 7.92 (2.69) | <0.001 |
| Episodic memory score, mean (SD) | 4.11 (1.55) | 3.41 (1.70) | <0.001 |

Notes: SD, standard deviations.

**Table S3. Association between childhood friendship status and cognitive ageing trajectory in middle-aged and older adults in imputed dataset**

| Fixed effect | β (95% CI) |  |  |  |
| --- | --- | --- | --- | --- |
|  | Model 1^a^ | Model 2^b^ | Model 3^c^ | Model 4^d^ |
| **Total cognitive function** |  |  |  |  |
| Constant | 16.86 (16.55, 17.17)^***^ | 16.97 (16.66, 17.29)^***^ | 11.95 (11.59, 12.32)^***^ | 11.96 (11.58, 12.34)^***^ |
| Time | -0.34 (-0.36, -0.31)^***^ | -0.43 (-0.47, -0.39)^***^ | -0.43 (-0.47, -0.39)^***^ | -0.43 (-0.47, -0.39)^***^ |
| Childhood friendship (Ref: poor) |  |  |  |  |
| Fair | 0.88 (0.77, 0.99)^***^ | 0.77 (0.63, 0.90)^***^ | 0.20 (0.08, 0.32)^**^ | 0.19 (0.07, 0.31)^**^ |
| Good | 1.48 (1.37, 1.59)^***^ | 1.29 (1.16, 1.42)^***^ | 0.37 (0.25, 0.49)^***^ | 0.33 (0.21, 0.45)^***^ |
| Childhood friendship (Ref: poor) *Time |  |  |  |  |
| Childhood friendship (fair)*Time | -- | 0.09 (0.04, 0.15)^**^ | 0.09 (0.04, 0.15)^**^ | 0.09 (0.04, 0.15)^**^ |
| Childhood friendship (good)*Time | -- | 0.15 (0.10, 0.21)^***^ | 0.15 (0.10, 0.21)^***^ | 0.15 (0.10, 0.21)^***^ |
| *p* value for trend | -- | <0.001 | <0.001 | <0.001 |
| **Mental intactness** |  |  |  |  |
| Constant | 10.49 (10.28, 10.70)^***^ | 10.45 (10.23, 10.66)^***^ | 7.39 (7.13, 7.65)^***^ | 7.37 (7.10, 7.64)^***^ |
| Time | -0.18 (-0.20, -0.17)^***^ | -0.16 (-0.19, -0.12)^***^ | -0.16 (-0.19, -0.12)^***^ | -0.16 (-0.19, -0.12)^***^ |
| Childhood friendship (Ref: poor) |  |  |  |  |
| Fair | 0.48 (0.41, 0.56)^***^ | 0.54 (0.44, 0.64)^***^ | 0.20 (0.11, 0.29)^***^ | 0.19 (0.10, 0.29)^***^ |
| Good | 0.81 (0.73, 0.88)^***^ | 0.86 (0.77, 0.96)^***^ | 0.32 (0.23, 0.41)^***^ | 0.30 (0.21, 0.39)^***^ |
| Childhood friendship (Ref: poor) *Time |  |  |  |  |
| Childhood friendship (fair)*Time | -- | -0.04 (-0.08, 0.01)^†^ | -0.04 (-0.08, 0.01)^†^ | -0.04 (-0.08, 0.01)^†^ |
| Childhood friendship (good)*Time | -- | -0.04 (-0.08, 0.00)^†^ | -0.04 (-0.08, 0.00)^†^ | -0.04 (-0.08, 0.00)^†^ |
| *p* value for trend | -- | 0.097 | 0.097 | 0.097 |
| **Episodic memory** |  |  |  |  |
| Constant | 6.31 (6.16, 6.46)^***^ | 6.45 (6.30, 6.60)^***^ | 4.58 (4.39, 4.77)^***^ | 4.61 (4.41, 4.81)^***^ |
| Time | -0.15 (-0.16, -0.14)^***^ | -0.27 (-0.30, -0.25)^***^ | -0.27 (-0.30, -0.25)^***^ | -0.27 (-0.30, -0.25)^***^ |
| Childhood friendship (Ref: poor) |  |  |  |  |
| Fair | 0.38 (0.33, 0.44)^***^ | 0.24 (0.17, 0.31)^***^ | 0.01 (-0.05, 0.08) | 0.01 (-0.05, 0.08) |
| Good | 0.65 (0.60, 0.70)^***^ | 0.44 (0.38, 0.50)^***^ | 0.06 (0.00, 0.13)^*^ | 0.04 (-0.02, 0.11) |
| Childhood friendship (Ref: poor)*Time |  |  |  |  |
| Childhood friendship (fair)*Time | -- | 0.13 (0.10, 0.16)^***^ | 0.13 (0.10, 0.16)^***^ | 0.13 (0.10, 0.16)^***^ |
| Childhood friendship (good)*Time | -- | 0.19 (0.16, 0.22)^***^ | 0.19 (0.16, 0.22)^***^ | 0.19 (0.16, 0.22)^***^ |
| *p* value for trend | -- | <0.001 | <0.001 | <0.001 |

Note: ^†^ 0.05 ≤ *p* < 0.1; ^*^ *p* < 0.05; ^**^ *p* < 0.01; ^***^ *p* < 0.001

--: Not included in model.

^a^ Model 1 was adjusted for age and sex.

^b^ Model 2 was adjusted as per Model 1 plus with the interaction term of time and childhood friendship.

^c^ Model 3 was adjusted as per Model 2 plus with sociodemographic factors (residence, marital status, educational level, and household expenditure per capita).

^d^ Model 4 was adjusted as per Model 3 plus with childhood conditions (childhood family financial situation, childhood first hukou, childhood health, and adverse childhood experiences).

**Table S4. Association between childhood friendship and the level of cognitive function among middle-aged and older adults**

| Fixed effect | β (95% CI) |  |  |
| --- | --- | --- | --- |
|  | Model 1^a^ | Model 2^b^ | Model 3^c^ |
| **Total cognition** |  |  |  |
| Constant | 15.37 (14.86, 15.88)^***^ | 11.83 (11.16, 12.50)^***^ | 12.03 (11.33, 12.73)^***^ |
| Time | -0.21 (-0.23, -0.19)^***^ | -0.22 (-0.26, -0.18)^***^ | -0.22 (-0.25, -0.18)^***^ |
| Childhood Friendship (Ref: poor) |  |  |  |
| Fair | 0.71 (0.51, 0.91)^***^ | 0.42 (0.24, 0.60)^***^ | 0.40 (0.22, 0.58)^***^ |
| Good | 1.23 (1.05, 1.41)^***^ | 0.64 (0.46, 0.82)^***^ | 0.61 (0.43, 0.79)^***^ |
| *p* value for trend | <0.001 | <0.001 | <0.001 |
| **Mental intactness** |  |  |  |
| Constant | 9.48 (9.15, 9.81)^***^ | 7.14 (6.69, 7.59)^***^ | 7.21 (6.75, 7.68)^***^ |
| Time | -0.24 (-0.26, -0.22)^***^ | -0.24 (-0.26, -0.22)^***^ | -0.24 (-0.26, -0.22)^***^ |
| Childhood Friendship (Ref: poor) |  |  |  |
| Fair | 0.39 (0.27, 0.51)^***^ | 0.23 (0.11, 0.35)^***^ | 0.22 (0.10, 0.34)^***^ |
| Good | 0.67 (0.55, 0.79)^***^ | 0.32 (0.20, 0.44)^***^ | 0.31 (0.19, 0.43)^***^ |
| *p* value for trend | <0.001 | <0.001 | <0.001 |
| **Episodic memory** |  |  |  |
| Constant | 6.00 (5.73, 6.27)^***^ | 4.73 (4.36, 5.10)^***^ | 4.85 (4.46, 5.24)^***^ |
| Time | 0.03 (0.01, 0.05)^**^ | 0.03 (0.01, 0.05)^**^ | 0.03 (0.01, 0.05)^*^ |
| Childhood Friendship (Ref: poor) |  |  |  |
| Fair | 0.33 (0.23, 0.43)^***^ | 0.19 (0.09, 0.29)^***^ | 0.19 (0.09, 0.29)^***^ |
| Good | 0.57 (0.47, 0.67)^***^ | 0.32 (0.22, 0.42)^***^ | 0.31 (0.21, 0.41)^***^ |
| *p* value for trend | <0.001 | <0.001 | <0.001 |

^†^ 0.05 ≤ *p* < 0.1; ^*^ *p* < 0.05; ^**^ *p* < 0.01; ^***^ *p* < 0.001

^a^ Model 1 was adjusted for age and gender.

^b^ Model 2 was adjusted as model 1 plus socio-demographic factors (residence, marital status, educational level and household consumption per capita).

^c^ Model 3 was adjusted as model 2 plus childhood conditions (childhood family financial situation, childhood first hukou, childhood health, adverse childhood experiences).

**Table S5.** **Interaction effects of childhood friendship status and potential moderators on the level of cognitive function in middle-aged and older adults**

| Model | Total cognitive function, β (95% CI) | Mental intactness,  β (95% CI) | Episodic memory,  β (95% CI) |
| --- | --- | --- | --- |
| **Model 1^a^** |  |  |  |
| Childhood friendship (Ref: poor) |  |  |  |
| Fair | 0.29 (0.06, 0.52)^*^ | 0.15 (-0.01, 0.30)^†^ | 0.15 (0.02, 0.28)^*^ |
| Good | 0.49 (0.26, 0.72)^***^ | 0.19 (0.04, 0.35)^*^ | 0.30 (0.17, 0.43)^***^ |
| Female (Ref: male) | -0.33 (-0.64, -0.03)^*^ | -0.54 (-0.74, -0.34)^***^ | 0.22 (0.05, 0.39)^*^ |
| Childhood friendship (fair) × Female | 0.27 (-0.10, 0.63) | 0.18 (-0.07, 0.42) | 0.09 (-0.12, 0.29) |
| Childhood friendship (good) × Female | 0.29 (-0.06, 0.64) | 0.27 (0.04, 0.50)^*^ | 0.02 (-0.17, 0.21) |
| **Model 2^a^** |  |  |  |
| Childhood friendship (Ref: poor) |  |  |  |
| Fair | 0.76 (0.17, 1.36)^*^ | 0.66 (0.26, 1.06)^**^ | 0.09 (-0.24, 0.43) |
| Good | 0.86 (0.24, 1.47)^**^ | 0.57 (0.16, 0.98)^**^ | 0.29 (-0.05, 0.63)^†^ |
| Educational level (Ref: illiterate) |  |  |  |
| Some primary school | 2.06 (1.50, 2.61)^***^ | 1.47 (1.10, 1.83)^***^ | 0.60 (0.29, 0.91)^***^ |
| Finished primary school | 2.53 (1.98, 3.07)^***^ | 1.79 (1.43, 2.15)^***^ | 0.76 (0.46, 1.06)^***^ |
| Higher than primary school | 3.42 (2.88, 3.95)^***^ | 2.35 (1.99, 2.70)^***^ | 1.09 (0.79, 1.39)^***^ |
| Childhood friendship (fair) × Some primary school | -0.47 (-1.18, 0.24) | -0.48 (-0.95, 0.00)^*^ | 0.03 (-0.37, 0.43) |

| Childhood friendship (fair) × Finished primary school | -0.31 (-0.99, 0.37) | -0.41 (-0.86, 0.05)^†^ | 0.11 (-0.27, 0.49) |
| --- | --- | --- | --- |

| Childhood friendship (fair) × Higher than primary school | -0.4 (-1.06, 0.27) | -0.54 (-0.98, -0.10)^*^ | 0.16 (-0.21, 0.53) |
| --- | --- | --- | --- |

| Childhood friendship (good) × Some primary school | -0.54 (-1.27, 0.19) | -0.43 (-0.91, 0.06)^†^ | -0.12 (-0.52, 0.29) |
| --- | --- | --- | --- |

| Childhood friendship (good) × Finished primary school | -0.29 (-0.99, 0.40) | -0.23 (-0.69, 0.23) | -0.06 (-0.45, 0.32) |
| --- | --- | --- | --- |
| Childhood friendship (good) × Higher than primary school | -0.16 (-0.84, 0.51) | -0.29 (-0.74, 0.15) | 0.13 (-0.24, 0.51) |
| **Model 3^a^** |  |  |  |
| Childhood friendship (Ref: poor) |  |  |  |
| Fair | 0.58 (0.21, 0.95)^**^ | 0.22 (-0.03, 0.46)^†^ | 0.37 (0.16, 0.57)^***^ |
| Good | 0.49 (0.14, 0.84)^**^ | 0.15 (-0.08, 0.39) | 0.33 (0.14, 0.53)^***^ |
| Adverse childhood experiences (Ref: 0) |  |  |  |
| 1 | -0.17 (-0.57, 0.24) | -0.16 (-0.43, 0.11) | -0.02 (-0.24, 0.21) |
| 2 | 0.01 (-0.41, 0.44) | -0.09 (-0.37, 0.19) | 0.09 (-0.14, 0.33) |
| ≥3 | -0.32 (-0.79, 0.14) | -0.4 (-0.71, -0.09)^*^ | 0.08 (-0.18, 0.34)^*^ |

| Childhood friendship (fair) × ACEs (1) | -0.10 (-0.59, 0.38) | 0.04 (-0.28, 0.36) | -0.13 (-0.40, 0.14) |
| --- | --- | --- | --- |

| Childhood friendship (fair) × ACEs (2) | -0.49 (-1.01, 0.02)^†^ | -0.15 (-0.49, 0.19) | -0.35 (-0.63, -0.06) |
| --- | --- | --- | --- |

| Childhood friendship (fair) × ACEs (≥3) | -0.21 (-0.78, 0.36) | 0.09 (-0.29, 0.47) | -0.30 (-0.61, 0.02)^†^ |
| --- | --- | --- | --- |

| Childhood friendship (good) × ACEs (1) | 0.17 (-0.28, 0.63) | 0.16 (-0.14, 0.46) | 0.02 (-0.23, 0.28) |
| --- | --- | --- | --- |
| Childhood friendship (good) × ACEs (2) | 0.07 (-0.42, 0.56) | 0.16 (-0.17, 0.48) | -0.07 (-0.34, 0.21) |
| Childhood friendship p (good) × ACEs (≥3) | 0.36 (-0.19, 0.90) | 0.41 (0.05, 0.77)^*^ | -0.04 (-0.35, 0.26) |

Note: ACEs, adverse childhood experiences.

^†^ 0.05 ≤ *p* < 0.1; ^*^ *p* < 0.05; ^**^ *p* < 0.01; ^***^ *p* < 0.001

^a^ Model was adjusted for sociodemographic factors and childhood conditions.

**References**

1. Lin L, Wang HH, Lu C, Chen W, Guo VY. Adverse Childhood Experiences and Subsequent Chronic Diseases Among Middle-aged or Older Adults in China and Associations With Demographic and Socioeconomic Characteristics. JAMA Netw Open. 2021;4(10):e2130143.

2. Xie J, Liao J, Zhang J, Gu J. Association between rural-to-urban migration and the cognitive aging trajectories of older Chinese adults: results from a prospective cohort analysis. BMC Geriatr. 2020;20(1):360.

3. Huang W, Zhou Y. Effects of education on cognition at older ages: evidence from China's Great Famine. Soc Sci Med. 2013; 98:54-62.

4. Huang W, Lei X, Ridder G, Strauss J, Zhao Y. Health, Height, Height Shrinkage, and SES at Older Ages: Evidence from China. Am Econ J Appl Econ. 2013;5(2):86-121.

5. Lei X, Hu Y, McArdle JJ, Smith JP, Zhao Y. Gender Differences in Cognition among Older Adults in China. J Hum Resour. 2012;47(4):951-971.
